# Temporal oculomotor inhibition of return and spatial facilitation of return in a visual encoding task

**DOI:** 10.3389/fpsyg.2013.00400

**Published:** 2013-07-02

**Authors:** Steven G. Luke, Joseph Schmidt, John M. Henderson

**Affiliations:** Visual Cognition Laboratory, Institute for Mind and Brain and Department of Psychology, University of South CarolinaColumbia, SC, USA

**Keywords:** inhibition of return, facilitation of return, eye movement control, saccadic momentum, foraging facilitator

## Abstract

Oculomotor inhibition of return (O-IOR) is an increase in saccade latency prior to an eye movement to a recently fixated location compared to other locations. It has been proposed that this temporal O-IOR may have spatial consequences, facilitating foraging by inhibiting return to previously attended regions. In order to test this possibility, participants viewed arrays of objects and of words while their eye movements were recorded. Temporal O-IOR was observed, with equivalent effects for object and word arrays, indicating that temporal O-IOR is an oculomotor phenomenon independent of array content. There was no evidence for spatial inhibition of return (IOR). Instead, spatial facilitation of return was observed: participants were significantly more likely than chance to make return saccades and to re-fixate just-visited locations. Further, the likelihood of making a return saccade to an object or word was contingent on the amount of time spent viewing that object or word before leaving it. This suggests that, unlike temporal O-IOR, return probability is influenced by cognitive processing. Taken together, these results are inconsistent with the hypothesis that IOR functions as a foraging facilitator. The results also provide strong evidence for a different oculomotor bias that could serve as a foraging facilitator: saccadic momentum, a tendency to repeat the most recently executed saccade program. We suggest that models of visual attention could incorporate saccadic momentum in place of IOR.

## INTRODUCTION

Inhibition of return (IOR) is a delay in response time to a target appearing at a previously attended location compared to a new location (Posner and Cohen, 1984). Although most paradigms used to study IOR focus on covert attention, IOR can also influence overt attention (eye movements). Oculomotor IOR (O-IOR) is an increase in saccade latencies when the eyes move back to a previously fixated location compared to a new control location (Klein and Hilchey, 2011). O-IOR, measured as an increase in fixation duration prior to a return saccade, has been reported in both simple visual tasks involving eye movements (Hooge and Frens, 2000; Ludwig et al., 2009; Farrell et al., 2010) and complex visual tasks such as searching for an object in a scene (Klein and MacInnes, 1999; MacInnes and Klein, 2003; Hooge et al., 2005; Thomas et al., 2006; Dodd et al., 2009; Smith and Henderson, 2011a, b), scene memorization and free viewing (Hooge et al., 2005; Smith and Henderson, 2009), and reading (Rayner et al., 2003; Weger and Inhoff, 2006; Henderson and Luke, 2012).

It has been proposed that O-IOR may have spatial consequences, inhibiting the return of the eyes to previously attended regions and thereby decreasing the probability of re-fixating those regions. Such a mechanism would increase the probability of fixating new locations, and thereby act as a *foraging facilitator*, increasing the likelihood of successful search by preventing attention from repeatedly returning to the same locations (Klein, 1988; Klein and MacInnes, 1999). This foraging facilitator hypothesis has prompted the incorporation of spatial IOR into many computational models of visual attention (Itti and Koch, 2001; Parkhurst et al., 2002; Rao et al., 2002; Pomplun et al., 2003; Navalpakkam and Itti, 2005; Wolfe, 2007; Sun et al., 2008; Zelinsky, 2008). In these models spatial O-IOR is typically realized as inhibitory tagging of the just-fixated location. The purpose of this tagging is to drive the eyes through the visual scene by ensuring that attention is not confined to one or a few particularly salient regions. In some models, this inhibitory tagging is permanent (e.g., Zelinsky, 2008) but in most it is temporary (e.g., Itti and Koch, 2001; Wolfe, 2007; Sun et al., 2008).

However, while the evidence for temporal O-IOR (a delay before returning) is robust, the evidence for spatial O-IOR (decreased probability of returning) is less so. In the literature on visual scenes, some researchers claim to have observed a decreased probability of returning to a previously fixated location (Klein and MacInnes, 1999), while others have observed facilitation of return, in that eye movements to previous locations are more likely than many other eye movements (Hooge et al., 2005; Smith and Henderson, 2009, 2011a, b; Wilming et al., 2013). Likewise, in reading the evidence is mixed. Weger and Inhoff (2006) present some evidence for spatial IOR in reading, but because they report no evidence for temporal IOR in the reading task their results are difficult to interpret. Both Rayner et al. (2003) and Henderson and Luke (2012) report significant temporal IOR in reading, but found no evidence for an associated decrease in the likelihood of making return saccades. Without consistent evidence for both a delay before returning *and* a decreased probability of returning, the foraging facilitator hypothesis may have to be re-evaluated. One of the purposes of the present study is to directly explore whether temporal O-IOR has spatial consequences in both reading and scene viewing, as predicted by the foraging facilitator hypothesis.

By far the most frequently occurring eye movements are saccades that are a repeat of the previously executed saccade program (Hooge et al., 2005; Smith and Henderson, 2009, 2011a, b). Smith and Henderson (2009) referred to this tendency to repeat saccade programs as *saccadic momentum*, and suggested that previous findings apparently demonstrating spatial O-IOR can be attributed not to a decreased probability of going back, but to an increased probability of going forward. Another purpose of the present research is therefore to further investigate saccadic momentum.

In the present study, we presented participants with arrays of objects and of words (object names). Using these arrays instead of visual scenes and text eliminated some potentially confounding factors. For example, some previous O-IOR studies employed highly complex visual scenes as stimuli, making it difficult to determine specifically what object was being fixated, as objects and features in real scenes tend to overlap. This overlap could potentially inflate estimates of return probability. There was no overlap in the arrays presented here. Whereas in reading the words are not overlapping, they appear in sentences and paragraphs that may lead to processing difficulty that forces readers to override IOR as they attempt to integrate different words into a coherent linguistic representation, thereby obscuring any spatial component of IOR. In arrays of words there is no need for any linguistic integration and so the possibility of processing difficulty or confusion should be reduced. Presenting arrays of words and objects in the same experiment will also make the two tasks more directly comparable, thereby permitting some conclusions to be made about similarities and differences in how O-IOR operates in scene viewing and reading. Using these arrays also had the advantage of greatly simplifying the analysis and interpretation of results. Previous studies investigating O-IOR using scenes or text have relied on complex transformations of the data, which can make results difficult to interpret. The use of arrays in the present study should help to reduce the ambiguity associated with such manipulations and clarify the role of O-IOR in guiding visual attention.

## MATERIALS AND METHODS

### PARTICIPANTS

Twelve participants from the University of South Carolina community completed the experiment. All participants were native English speakers with 20/20 corrected or uncorrected vision.

### APPARATUS

Eye movements were recorded via an SR Research Eyelink 1000 eye tracker (spatial resolution of 0.01°) sampling at 1000 Hz. Subjects were seated 90 cm away from a 20-in monitor, so that objects and words subtended approximately 3.3° × 2.5° of visual angle. Head movements were minimized with chin and head rest. Although viewing was binocular, eye movements were recorded from the right eye. The experiment was controlled with SR Research Experiment Builder software.

### MATERIALS

**FIGURE 1:**
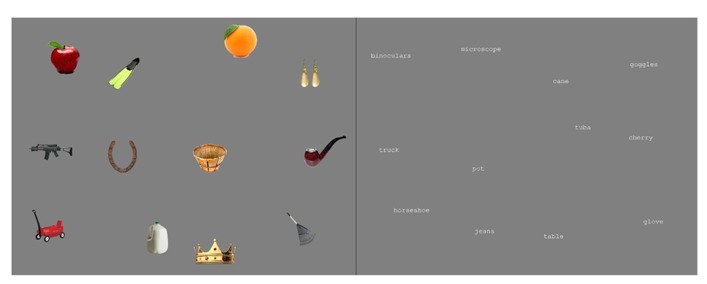
**Examples of the arrays used in the experiment**.

Each trial consisted of 12 objects or 12 words (object names) presented in a 4 × 3 array. The screen was divided into twelve 200 × 200 pixel (6.36° × 6.36°) regions, and one object or word appeared in each region. Each object or word was a 133 × 100 pixel (4.23° × 3.18°) image, and the center of each was jittered randomly (0–33 pixels × 0–50 pixels offset) within its respective region. For the critical trials, 15 arrays of objects and 15 arrays of words were created. An additional eight arrays were created for inclusion in the memory test at the end of the experiment. Two example arrays are shown in **Figure 1**

### PROCEDURE

Participants were told that they would be viewing sets of images or words on a computer screen while their eye movements were recorded. They were further instructed to view the arrays in preparation for a memory test that would be administered at the end of the session. This task was chosen to maximize the number of fixations, to make the number of fixations comparable across the different array types, and to ensure that most objects and words were fixated. Each trial began with a gaze trigger, a black circle presented in the center of the screen. Once a stable fixation was detected on the gaze trigger, the array was presented. The participant viewed the array for 10 s after which the array was removed from the screen. Then a new gaze trigger appeared and the next trial began. Arrays were presented in a random order for each participant.

## RESULTS

### SACCADE-BASED ANALYSES

The first set of analyses we performed were designed to reproduce the findings from other studies of IOR. Following Hooge et al. (2005), saccades longer than 12° and shorter than 1° and the fixations preceding these saccades were excluded from the data. The data were analyzed using linear mixed models in R (R Development Core Team, 2012), with participant as a random effect. *p*-Values for these analyses were obtained using Markov Chain Monte Carlo sampling. In these analyses, return saccades were defined as saccades that were similar in amplitude to the previous saccade (±4°) and in approximately the opposite direction (180 ± 30°), again following Hooge et al. (2005). In addition, Array Type (Objects or Words) was also included in the analyses as a predictor. Because the amplitude of saccades was not controlled directly, saccade amplitude was also included in the analyses as a covariate.

#### Temporal IOR

Significant temporal IOR was observed (Coeff. = 0.054, SE = 0.024, *t* = 2.24, *p* = 0.025). There was also a significant effect of array type, with shorter fixations on words than on objects for non-return fixations (Coeff. = -0.081, SE = 0.01, *t* = -8.13, *p* = 0.0001; effect size = 18 ms). An interaction between Return and Array Type indicated that the effect of array type disappeared for fixations preceding return saccades (Coeff. = 0.11, SE = 0.033, *t* = 3.27, *p* = 0.0011). Because of this difference in the durations of the baseline non-return fixations, the magnitude of the temporal IOR for object arrays was reduced compared to word arrays (13 vs. 39 ms, respectively). The effect of saccade amplitude on fixation duration was also significant, with longer fixations preceding larger saccades (Coeff. = 0.013, SE = 0.0012, *t* = 10.58, *p* = 0.0001), but the magnitude of the temporal IOR was not contingent on saccade size (*p* = 0.23).

#### Spatial IOR

**FIGURE 2:**
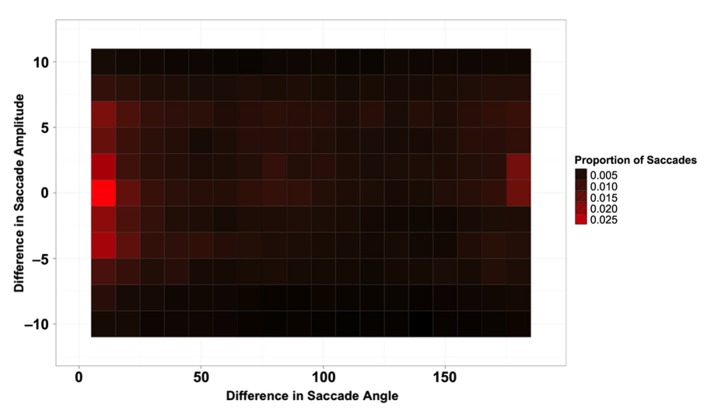
**Proportion of saccades as a function of the difference in amplitude (*y*-axis) and angle (*x*-axis) between the previous saccade and the current one**.

**Figure 2** shows the proportion of saccades as a function of the difference in both direction (*x*-axis) and amplitude (*y*-axis) between the current and previous saccade. **Figure 2** shows clear evidence for saccadic momentum; a large probability peak is apparent for saccades with close to zero change in angle and amplitude. Another somewhat smaller peak is apparent for return saccades, which had a minimal change in amplitude but a 180° change in direction from the previous saccade.

In order to confirm these observations statistically, saccades that differed in amplitude from the preceding saccade by more than ±2° were excluded from the data. The remaining saccades were grouped into 30° angular difference bins. The first bin (0–30°) represents forward momentum saccades that differed in angle from the preceding saccade by no more than 30°, and the final bin (150–180°) represents return saccades that differed in angle from the preceding saccade by no less than 150°. Other bins represent saccades that turned to the left or right by varying amounts.

**FIGURE 3:**
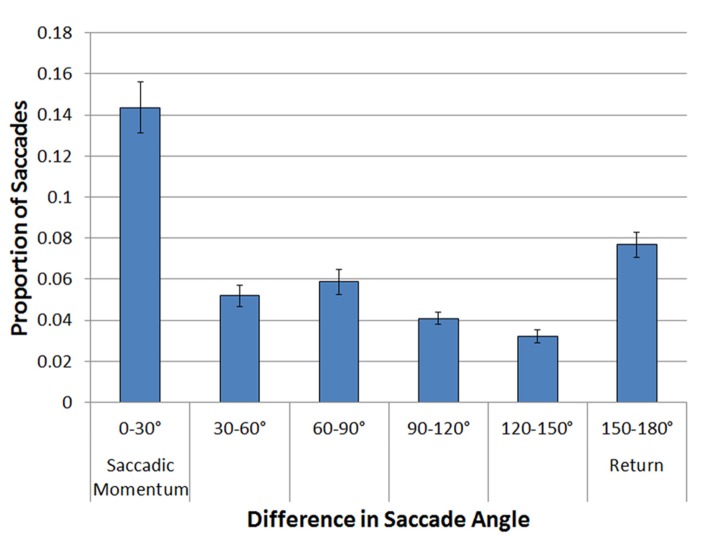
**Proportion of saccades as a function of the difference in angle between the current and the previous saccade, holding amplitude from previous to current saccade constant**.

A logit mixed effects model with participant as a random effect was performed on these data, comparing each of the 30° bins to the Return Saccade bin (150–180°). The probability of repeating the same saccade program (the saccadic momentum condition) was significantly higher than the probability of making a return saccade (Coeff = 0.85, SE = 0.052, *z* = 16.47, *p* = 0.0001). Return saccades were significantly more frequent than all other types of saccades (all *p*s < 0.001). No effects or interactions involving Array Type were significant (all *p*s < 0.6). These results are illustrated in **Figure 3.**

#### Summary

While temporal IOR was observed, with longer fixations preceding return saccades, there was no evidence that IOR acts as a foraging facilitator by decreasing return probability. On the contrary, with the exception of saccades that continued in the same direction as the preceding saccade, return saccades were more frequent than saccades in all other directions. Thus, these analyses provide evidence for spatial saccadic momentum, but no evidence for spatial IOR.

### LOCATION-BASED ANALYSES

The analyses reported above reproduce the results of previous studies that investigated IOR using free viewing of complex visual scenes (Hooge et al., 2005; Smith and Henderson, 2009, 2011a, b). They indicate that participants were most likely to continue moving their eyes in the direction they had moved in the preceding saccade. Of the remaining directions other than forward, participants were most likely to make return saccades that moved the eyes back in the direction they had come from.

In the analyses reported below, we defined return saccades based on the object or word to which the eyes are moving. A return saccade was defined as a saccade that took the eyes back from the currently fixated object or word (object N) to a previously fixated object or word (either object N-1 or N-2). As in the previous analyses, saccades longer than 12° and shorter than 1° and the fixations preceding these saccades were excluded from the data. The data were analyzed using mixed models in R, with participant as a random effect.

#### Temporal IOR

For object N-1, a significant effect of Return was observed; fixations preceding saccades to object N-1 were significantly longer than other fixations (Coeff. = 0.091, SE = 0.023, *t* = 4.06, *p* = 0.0001; effect size = 21 ms). There was also a significant effect of saccade amplitude (Coeff. = 0.048, SE = 0.0027, *t* = 17.52, *p* = 0.0001, effect size = 129 ms), indicating that fixation duration increased as saccade amplitude increased. A significant interaction of Return and Saccade Amplitude indicated that temporal IOR decreased as saccade amplitude increased, because the effect of saccade amplitude on fixation durations was weaker (67 ms) for return saccades (Coeff. = -0.024, SE = 0.0083, *t* = -2.93, *p* = 0.0034). The effect of Array Type was significant (Coeff. = -0.029, SE = 0.014, *t* = -2.03, *p* = 0.042; effect size = 10 ms), indicating that fixations were somewhat shorter on words than on objects. Array Type did not interact with Return (*p* = 0.14).

For object N-2, there was no effect of Return, nor did Return interact with the other predictors (all *p*s > 0.5). Effects of Saccade Amplitude and Array Type were observed as reported in the analysis for object N-1 (both *p*s < 0.001).

#### Spatial IOR

**FIGURE 4:**
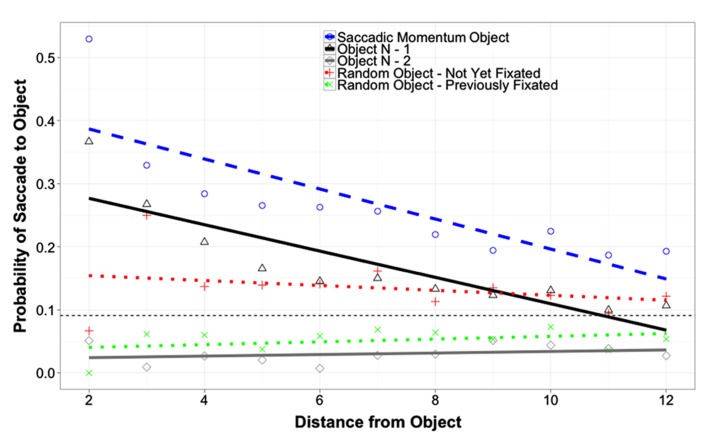
**Probability of saccade to an object as a function of object and of distance from that object (in degrees of visual angle).** The small horizontal dashed line represents the random chance probability of a saccade from the currently fixated object to any of the other objects in the array.

**Figure 4** shows the probability of returning to fixate an object as a function of object and of the distance between the current fixation and the object in question. Because the distance from the current fixation to the different objects was not controlled directly, this distance was included in these analyses as a covariate. Probabilities for three critical objects and two random control objects are plotted. The first object is the saccadic momentum object, which is the object the eyes would move to if they continued in the same direction as the last saccade. The second object is object N-1, and the third is object N-2. The first random control object is an object randomly selected from the pool of not-yet-fixated objects in the array that was not one of the three critical objects or the currently fixated object. The second random control object is an object randomly selected from the pool of previously fixated objects in the array that was not one of the three critical objects or the currently fixated object.

Two analyses were conducted on these data. The first compared the probability of saccades to object N-1 to the probability of saccades to each of the other four objects, while the second made the same comparisons for object N-2. The first analysis revealed a greater likelihood of saccades to the Saccadic Momentum control object than to object N-1 (Coeff. = 0.075, SE = 0.037, *t* = 2.04, *p* = 0.041). Saccades to object N-1 were significantly more likely than saccades to any of the three objects other than the Saccadic Momentum object (all *p*s < 0.001). The effect of distance was significant for object N-1 and the Saccadic Momentum Control Object (Coeff. = -0.022, SE = 0.0033, *t* = -6.7, *p* = 0.0001), but significant interactions between Object and Distance indicated that the effect of distance disappeared for object N-2 and the two Random control objects (all *p*s < 0.005). These interactions also indicate that the difference in probability of re-fixation between objects N-1 and N-2 as well as the two control objects decreased as distance increased. The effect of array type was not significant, nor did it interact with any other predictors (all *p*s > 0.24). A second analysis revealed that the likelihood of re-fixating object N-2 was significantly less than all other objects (all *p*s < 0.001) except the random previously fixated object (*p* = 0.66). As no temporal O-IOR was observed for object N-2, this cannot be interpreted as spatial O-IOR. Instead, this pattern of results probably reflects information seeking directed by top-down processes. Analysis two also reproduced the other effects and interactions from the first analysis.

#### Cognitive control of return saccades

**FIGURE 5:**
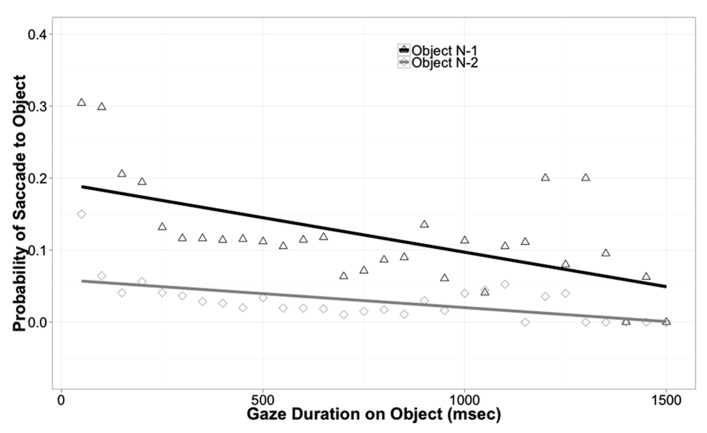
**Probability of saccade back to an object as a function of object and of previous gaze duration on that object (i.e., how long the object was viewed before the eyes left it)**.

In reading, return saccades are known to be influenced by a variety of cognitive factors, indicating that they are under cognitive control (Rayner, 1998). To explore whether return saccades are under cognitive control in the current experiment, we used gaze duration, the time spent viewing an object before leaving it, as a predictor of return probability. If return probability decreases as gaze duration increases, this would suggest that participants make return saccades in order to process an object more fully. All saccades in the data set that took the eyes to a new object were coded as either a return saccade (if the new object was object N-1) or a non-return saccade. Logit mixed models were then fit to this data, with Array Type and Gaze Duration as predictors and participant as a random effect. For object N-1, Gaze Duration was a significant predictor of return probability; objects with shorter gaze durations were more likely to be the target of return saccades (Coeff. = -0.8, SE = 0.069, *z* = -11.68, *p* = 0.0001). Performing the same analysis for object N-2 revealed a significant effect of Gaze Duration, again indicating that shorter gaze durations were associated with higher return probability (Coeff. = -0.76, SE = 0.14, *z* = -5.28, *p* < 0.0001). These effects are illustrated in **Figure 5.** The effect of Array Type was again non-significant (*p* = 0.9). These analyses strongly show that the likelihood of return is related to processing requirements. When the eyes moved away from an object or word quickly, a return saccade was more likely, but when the eyes fixated an object for a longer time, allowing more complete visual processing, return saccades became less likely. Thus, these analyses suggest that return saccades are under cognitive control.

#### Summary

Significant temporal IOR was observed for saccades to object N-1 but not to object N-2. At the same time, significant spatial facilitation of return for object N-1 was observed. Thus, while these analyses provide additional evidence for spatial saccadic momentum, they again provide no evidence for spatial IOR. We also observed evidence for cognitive control of eye movements; the eyes were significantly more likely to return to a previously fixated object if they had spent less time on that object before leaving.

## DISCUSSION

The foraging facilitator hypothesis predicts that return saccades, which take the eyes back to a previously fixated location, should be both less likely to occur than other saccades and, when they do occur, should be preceded by a delay. In the present study, this delay before returning (O-IOR) was observed consistently, but no evidence was found for a decreased probability of return. Instead, significant facilitation of return was observed: return saccades were more likely than saccades in any other direction (except forward). This was true even though the eyes were unambiguously returning back to the object they had just left. In order for IOR to act as a foraging facilitator, it must first and foremost inhibit shifts of attention to the just-fixated location. Otherwise, attention could cycle continuously between the two most salient locations in a stimulus. The results of the present study show clearly that return saccades to the just-fixated location are not inhibited, but are instead facilitated compared to most other locations. These results are thus highly problematic for the foraging facilitator hypothesis. It is possible that task demands in the current study may have altered return saccade probability; the need to remember the objects and words in the arrays may have increased the likelihood of making return saccades (cf. Zelinsky et al., 2011), and that fewer return saccades would have been observed in a visual search task where distractor objects do not need to be committed to memory. Even so, any spatial effects of O-IOR should have been detectable in the current study. Temporal O-IOR was observed in the present study and has been observed in other studies that employed non-search tasks (Hooge et al., 2005; Smith and Henderson, 2009), so it is clear that O-IOR does influence eye movement behavior in non-search tasks, including the present one. The presence of these cross-task temporal O-IOR effects show that temporal O-IOR is relatively insensitive to task (cf. Henderson and Luke, 2012), and so one would expect that any spatial consequences of O-IOR would be observable regardless of task as well. Further, spatial O-IOR has been nearly ubiquitously incorporated into models of visual attention (Rao et al., 2002; Pomplun et al., 2003; Wolfe, 2007; Sun et al., 2008; Zelinsky, 2008), even those designed to model non-search behavior (Itti and Koch, 2001; Parkhurst et al., 2002; Navalpakkam and Itti, 2005), suggesting that spatial O-IOR should have been detectable regardless of task. In order to serve as a foraging facilitator, the spatial consequences of O-IOR must be strong enough to actually prevent the eyes from returning, and the results reported here and elsewhere show that this is not in fact the case. Thus, IOR is not an ideal mechanism to serve as a foraging facilitator.

The results of the present study suggest an alternative mechanism to replace IOR as a foraging facilitator: saccadic momentum, the tendency to repeat a saccade program and to continue moving the eyes in the same direction (Smith and Henderson, 2009). In all spatial analyses reported here the most frequent eye movement was one in approximately the same direction and with approximately the same amplitude as the previous saccade. Saccadic momentum has been observed repeatedly in other studies as well, establishing it as a robust and consistent bias of the oculomotor system (Hooge et al., 2005; Smith and Henderson, 2009, 2011a, b; Wilming et al., 2013). Functionally, a bias to move the eyes forward would serve the same purpose as a bias against returning, driving the eyes through the stimulus in a manner that would facilitate foraging. Given that saccadic momentum is observed so often and spatial O-IOR has proven difficult to establish empirically, the former seems to be a better candidate to serve as a foraging facilitator in models of visual attention.

Saccadic momentum could be incorporated into most current models of visual attention using a mechanism similar to the inhibitory tagging they currently employ. Instead of inhibiting the just-fixated object or location, excitation could be added to the saccadic momentum object or location. If the saccadic momentum location is excited sufficiently that it is more active than other locations, then there will be a constantly moving activation peak that draws attention away from currently and previously fixated locations, thereby driving attention through the stimulus.

Whatever the mechanism that drives attention through a stimulus, it is also abundantly clear that return saccades do occur, sometimes quite frequently. Smith and Henderson (2009), (2011a, b) have suggested that return saccades are under cognitive control, meaning that the likelihood of making a return saccade is modulated by ongoing processing, with greater stimulus complexity and/or processing difficulty leading to a greater likelihood of returning. This proposal is based on the observation that return saccades are more common for more complex stimuli, such as visual scenes, than for less complex stimuli. The current study provides additional evidence that return saccades are under cognitive control, and are initiated in order to more thoroughly process an object: objects with shorter gaze durations were more likely to be the target of return saccades. In other words, return saccades occur in the service of *information accrual.* This function of return saccades is well established in the reading literature. Reading has regressions, which increase in frequency as processing difficulty increases (e.g., syntactic parsing difficulty or infrequent words; Rayner, 1998). This is in spite of the fact that temporal IOR does occur in reading (Rayner et al., 2003; Henderson and Luke, 2012). Likewise, although temporal IOR was observed in the present study, return saccades still occurred. Further, saccades are sometimes initiated before processing is completed (Henderson and Pierce, 2008; Rayner et al., 2009; Luke et al., 2013), and as Smith and Henderson (2009, p. 1103) point out, “if the saccade programing system cannot be relied upon to hold fixation until adequate visual encoding has finished, a compensatory system which represents previously fixated locations and facilitates return saccades would be required.” The present study provides evidence for this compensatory system, in which return saccades are initiated in order to complete interrupted visual encoding.

Additional evidence in favor of a top-down system guiding information accrual comes from object N-2 and the two control objects. Saccades to objects that had been fixated previously (and that were not object N-1) were significantly less frequent than saccades to not-yet-fixated objects (see **Figure 4**). This reveals that the participants had a tendency to seek out new objects and to avoid old ones. The fact that no temporal O-IOR was observed for object N-2 indicates that this tendency was the result of top-down guidance and not the result of IOR.

Thus, models of visual attention and eye movement control should incorporate a top-down information accrual mechanism. This mechanism could tag locations that should be visited and inhibit locations when some information accrual threshold has been reached. It could also initiate return saccades when the threshold had not been reached prior to leaving an object. Adjustments to the information accrual mechanism could allow models to account for task-based differences in eye movement behavior (Henderson and Hollingworth, 1998; Castelhano et al., 2009; Luke et al., 2013).

It is interesting to note that in the present study the type of array had essentially no influence on temporal O-IOR. This observation reinforces the conclusion that O-IOR is indeed an oculomotor phenomenon that is stimulus-independent, and so is consistent with Henderson and Luke (2012), who showed an equivalent O-IOR effect in normal reading and in a mindless reading task in which all meaning was removed from the text. While further work is needed, the present study suggests that temporal O-IOR effects may be similar even for extremely different stimulus types such as real scenes and text.

## Conflict of Interest Statement

The authors declare that the research was conducted in the absence of any commercial or financial relationships that could be construed as a potential conflict of interest.
